# Recurrence of Papillary Thyroid Cancer: A Systematic Appraisal of Risk Factors

**DOI:** 10.1210/clinem/dgab836

**Published:** 2021-11-16

**Authors:** Hannah R Nieto, Caitlin E M Thornton, Katie Brookes, Albert Nobre de Menezes, Alice Fletcher, Mohammed Alshahrani, Merve Kocbiyik, Neil Sharma, Kristien Boelaert, Jean-Baptiste Cazier, Hisham Mehanna, Vicki E Smith, Martin L Read, Christopher J McCabe

**Affiliations:** Institute of Metabolism and Systems Research, University of Birmingham, Birmingham B15 2TT, UK; Institute of Metabolism and Systems Research, University of Birmingham, Birmingham B15 2TT, UK; Institute of Metabolism and Systems Research, University of Birmingham, Birmingham B15 2TT, UK; Centre for Computational Biology, University of Birmingham, Birmingham B15 2TT, UK; Institute of Cancer and Genomic Sciences, University of Birmingham, Birmingham B15 2TT, UK; Institute of Metabolism and Systems Research, University of Birmingham, Birmingham B15 2TT, UK; Institute of Metabolism and Systems Research, University of Birmingham, Birmingham B15 2TT, UK; Institute of Metabolism and Systems Research, University of Birmingham, Birmingham B15 2TT, UK; Institute of Cancer and Genomic Sciences, University of Birmingham, Birmingham B15 2TT, UK; Institute of Applied Health Research, University of Birmingham, Birmingham B15 2TT, UK; Centre for Computational Biology, University of Birmingham, Birmingham B15 2TT, UK; Institute of Cancer and Genomic Sciences, University of Birmingham, Birmingham B15 2TT, UK; Institute of Cancer and Genomic Sciences, University of Birmingham, Birmingham B15 2TT, UK; Institute of Metabolism and Systems Research, University of Birmingham, Birmingham B15 2TT, UK; Institute of Metabolism and Systems Research, University of Birmingham, Birmingham B15 2TT, UK; Institute of Metabolism and Systems Research, University of Birmingham, Birmingham B15 2TT, UK

**Keywords:** papillary, thyroid, recurrence, miRNA, TCGA

## Abstract

**Context:**

Thyroid cancer recurrence is associated with increased mortality and adverse outcomes. Recurrence risk is currently predicted using clinical tools, often restaging patients after treatment. Detailed understanding of recurrence risk at disease onset could lead to personalized and improved patient care.

**Objective:**

We aimed to perform a comprehensive bioinformatic and experimental analysis of 3 levels of genetic change (mRNA, microRNA, and somatic mutation) apparent in recurrent tumors and construct a new combinatorial prognostic risk model.

**Methods:**

We analyzed The Cancer Genome Atlas data (TCGA) to identify differentially expressed genes (mRNA/microRNA) in 46 recurrent vs 455 nonrecurrent thyroid tumors. Two exonic mutational pipelines were used to identify somatic mutations. Functional gene analysis was performed in cell-based assays in multiple thyroid cell lines. The prognostic value of genes was evaluated with TCGA datasets.

**Results:**

We identified 128 new potential biomarkers associated with recurrence, including 40 mRNAs, 39 miRNAs, and 59 genetic variants. Among differentially expressed genes, modulation of FN1, ITGα3, and MET had a significant impact on thyroid cancer cell migration. Similarly, ablation of miR-486 and miR-1179 significantly increased migration of TPC-1 and SW1736 cells. We further utilized genes with a validated functional role and identified a 5-gene risk score classifier as an independent predictor of thyroid cancer recurrence.

**Conclusion:**

Our newly proposed risk model based on combinatorial mRNA and microRNA expression has potential clinical utility as a prognostic indicator of recurrence. These findings should facilitate earlier prediction of recurrence with implications for improving patient outcome by tailoring treatment to disease risk and increasing posttreatment surveillance.

Thyroid cancer recurrence is associated with increased mortality (1, 2), and early detection of thyroid cancer recurrence has been shown to improve patient outcomes (3, 4). Currently, risk of recurrence is predicted using clinical tools that often restage patients after their cancer treatment. While this is useful in stratifying patients to the level of follow-up and degree of thyrotropin (thyroid stimulating hormone) suppression required postoperatively, it does not inform the surgeon and patient about risk of recurrence until after all treatment has been completed.

The primary management of thyroid neck recurrence remains surgical, with any microscopic residual disease managed via radioiodine if the malignancy is radioiodine avid (5). Distant metastases of differentiated thyroid cancer are often not radioiodine avid (4) and are particularly difficult to treat. If not amenable to surgery, radioiodine refractory disease can be considered for high-dose external beam radiotherapy (EBRT) (6), but this is considered much less effective. Sometimes radioresistant disease can be indolent and can be monitored (5). For rapidly progressive, symptomatic, or imminently threatening disease not amenable to local control, kinase inhibitor therapy can be considered (5), although this has a significant associated morbidity. Despite recent progress, most patients’ tumors eventually stop responding to kinase inhibitor therapies, and almost all patients have adverse events (7-10). Therapy targeted to individual patient genotype and phenotype may be key to future progress (11). Within this broader context, understanding which thyroid carcinomas are going to recur, and the functional reasons behind the recurrence, will be crucial to improving patient care.

For a malignancy to recur, the primary disease must evade treatment. Tumor cell dormancy has been postulated as a means by which cancer cells achieve this (12). The most common site of recurrence is the cervical lymph nodes (13), and metastatic dormancy may be a key component of how cancer cells survive but do not progress immediately in a metastatic environment (14). The identification of cancer cells in adequately treated patients lends support to the dormancy theory and could contribute to explaining how some thyroid cancer patients have a recurrence many years after their primary disease (15). An important concept then, is how dormant cancer cells “activate” to cause such recurrence.

There are multiple accompanying mechanisms which may contribute to thyroid cancer recurrence, including increased angiogenesis (16), escape from immune detection (17), and changes in the extracellular matrix (15). Investigating alterations in such pathways to identify significant biomarkers of thyroid cancer recurrence is important due to the significant morbidity and mortality associated with recurrence. Here, analysis of existing next-generation sequencing data was employed to investigate gene (mRNA/microRNA [miR]) expression changes and somatic mutational profiles associated with thyroid cancer recurrence. By performing in vitro investigations to determine the functional impact of these biomarkers, we aimed to discover how they might relate to the clinical outcome of thyroid cancer patients.

## Materials and Methods

### Patient Clinical Data and Bioinformatics

Bioinformatic analysis of potential clinical biomarkers for thyroid cancer recurrence was performed using 3 study arms: mRNA expression, somatic mutation, and miR expression (Fig. S1A (18)). Candidate biomarkers were validated by functional assessment to interrogate mechanisms of recurrence and significant output integrated to construct a prognosis risk model.

Total RNAseq data from the 501 thyroid cancer (THCA) samples described in The Cancer Genome Atlas (TCGA) (19) were analyzed, including 455 nonrecurrent and 46 recurrent tumor specimens (Table S1 and S2 (18)). In addition, 59 tumor/normal matched samples were also analyzed. Bioinformatic and statistical analyses were predominantly performed in the open-source software R (www.r-project.org). RNA expression analysis was performed with the TCGA RNA sequencing data (RNAseq by Expectation Maximization [RSEM] normalized RNA data files) through the FireHose portal (https://gdac.broadinstitute.org). The TCGA RNA sequencing (RNAseq) data have been upper quartile normalized. For each of the 20 532 genes, the absolute median differential expression between recurrent and nonrecurrent patients was calculated. The Mann-Whitney U value was calculated in R, and the genes were then ranked by median differential expression. MiR data from 502 THCA samples in TCGA were downloaded and normalized to reads per million counts. The median differential expression of each miR was conducted in the same way as the RNAseq data.

Access to TCGA variant call format (vcf) data files was granted after application via the National Institutes of Health CGHub portal to controlled access data, which were downloaded via GeneTorrent onto the University of Birmingham’s BlueBEAR HPC service (http://www.birmingham.ac.uk/bear). These files were annotated by Annovar (20) and exonic mutations filtered using Python by quality score and dbSNP (single nucleotide polymorphism database) (www.ncbi.nlm.nih.gov/snp), and then by a series of bioinformatic scoring tools (Fig. S1B (18)). In parallel, all patients who went on to have thyroid cancer recurrence had tumor and matched normal aligned sequence (bam) files downloaded. Variant calling, using Platypus (21), was performed on these data as a group analysis. Annovar annotation was again performed on the vcf file, and the annotated vcf was filtered using the same tools as the Mutect vcf analysis Filtering included removal of low-quality variants and those with synonymous mutations. In-silico mutation prediction was performed on both analyses using several bioinformatic tools: Sorting Intolerance From Tolerance (SIFT) score (22), Polymorphism Phenotyping version 2 (PolyPhen2) score (23), MutationTaster (24) and MutationAssessor (25) score. Scores were combined to identify the most harmful mutations.

### Patient Survival Characteristics

Receiver operating characteristic (ROC) curves were plotted in R, using the cutpointR package. Using the cutpoint generated, the patients were grouped into high risk and low risk of recurrence, for each individual gene. A Cox multivariate analysis was then run in R using the survival and survminer packages, with forest plots generated with ggplot2. Using a continuous variable multivariate analysis, the Cox regression coefficient was identified for each gene. This was then multiplied by the expression value (miR or RNAseq) for each patient, and the risk score for each gene was then combined to create a combined risk score. A ROC curve analysis and Kaplan-Meier curve was then generated for the combined risk score.

### Cell Culture

Three cell lines were cultured for cell line–based assays: the human differentiated papillary thyroid cancer TPC-1 cell line, the human anaplastic thyroid cancer SW1736 cell line, and the human de-differentiated thyroid cancer CAL62 cell line. The TPC-1 and SW1736 lines were kindly provided by Prof Rebecca Schweppe (University of Colorado; Colorado, USA). TPC-1 cells are derived from a moderately differentiated human papillary thyroid carcinoma, with mutational rearrangement on chromosome 10 causing the RET/PTC1 chimera (26, 27) with wild-type BRAF and RAS. The SW1736 cell line is derived from an anaplastic thyroid tumor and contains the BRAF^V600E^ mutation (28, 29). The CAL62 cell line was kindly provided by Prof. James Fagin (Memorial Sloan Kettering Cancer Center; New York, USA). CAL62 cells are derived from an anaplastic thyroid tumor and harbor a RAS mutation (KRAS G12R) (30). These cell lines were cultured in Roswell Park Memorial Institute (RPMI)-1640 media with L-glutamine (Life Technologies, ThermoFisher Scientific; Massachusetts, USA) supplemented with 1% Penicillin (105 U/L)/Streptomycin (100 mg/L) (Life Technologies) and 10% heat-inactivated fetal bovine serum (FBS) (Life Technologies). All cell lines were maintained at low passage, authenticated by short tandem repeat analysis (NorthGene), and tested for mycoplasma contamination (EZ-PCR; Geneflow). Profiling of the relative basal gene expression of candidate biomarkers across a panel of thyroid cancer cells is given (Fig. S2) (31).

### Nucleic Acids and Transfection

The following plasmids and expression vectors were transfected into the above thyroid cancer cell lines using transfection reagent TransIT-LT1 (LT1) (GeneFlow; Lichfield, UK) at a ratio of 3 μL to 1 μg DNA in Opti-MEM I reduced serum medium (Life Technologies): DICER1 (pCMV-flag; generously supplied by Prof. P Santisteban, the Autonomous University of Madrid, Spain), IMPDH2 (pCMV6-XL5; #SC119585; Origene Technologies; Rockville, MD, USA), PFKFB4 WT (CMV6-XL5; #SC110972; Origene Technologies; Rockville, MD, USA); FN1 (pPM-N-D-C-HA; #PV355032 abm; Vancouver, Canada), MET (CMV3-ORF; #HG10692-UT Sino Biological; Pennsylvania, USA). Patient mutations were recapitulated using the Quick-Change II XL site directed mutagenesis kit (Agilent Technologies; California, USA). Cell lines were transfected with various small interfering RNAs (siRNAs) using lipofectamine RNAiMAX (Invitrogen). One mL of Opti-MEM I reduced serum medium (Life Technologies) was mixed with 6 µL lipofectamine RNAiMAX and allowed to stand for 5 minutes. Then siRNA was added to the Opti-MEM mix to final concentrations of 100nM. For miR transfection and knockdown, 6 μL of lipofectamine RNAiMAX (Invitrogen) was added to 1 mL of Opti-MEM I reduced serum medium. The microRNA miR negative control mimic (CN-001000-01-05), miR-221-5p mimic (C-301163-01-0002), miR-486-5p inhibitor (IH-300746-05-0002), or miR-1179 inhibitor (IH-301320-01-0002) (Dharmacon, Colorado, USA) was then added to a final concentration of 100nM. Transfection and knockdown were confirmed with microRNA real time quantitative PCR (RTqPCR).

### Western Blotting

Western blotting was performed as previously described (32, 33) following DNA plasmid transfection or siRNA transfection as indicated. Blots were probed with specific antibodies to inosine monophosphate dehydrogenase 2 (IMPDH2), 6-phosphofructo-2-kinase/fructose-2,6-bisphosphatase 4 (PFKFB4), DICER1, integrin α3 (ITGα3), and fibronectin 1 (FN1), relative to the expression of β-actin.

### Cell Proliferation Assays

Cells were seeded into 96-well plates in 100 µL of media 24 hours after transfection with 6 replicates of each condition. After a further 24 hours, 20 µL of CellTiter 96 AQueous One Solution Cell Proliferation Assay solution (Promega, UK) was added to each well. Colorimetric absorbance was measured at 490 nM on the Wallac 1420 Victor 3 Multilabel Counter plate reader (Perkin-Elmer). Background absorbance was subtracted from media-only wells.

### Cell Invasion Assays

Four hours after transfection, normal (10% FBS) cell medium was replaced with 2% FBS media. The following day, Corning BioCoat growth factor reduced Matrigel invasion chambers (Corning; New York, USA) were warmed and rehydrated. Initial experiments evaluated optimum cell density for each line (data not shown), and invasion assays were carried out as we have described previously (34), before Eosin Y solution (Sigma-Aldrich) staining and being photographed on an EVOS XL Core Imaging System (ThermoFisher Scientific).

### Cell Migration Assays

Cells were seeded and then transfected 24 hours later with the gene of interest. A further 24 to 48 hours later, media were changed to 2% FBS to control for cell proliferation and then a linear vertical scratch was made through the center of the well with a 200 µL pipette tip. Photographs were taken at the same point along the scratch wound on the EVOS XL Core Imaging System (ThermoFisher Scientific) at 0, 4, 8, and 24 hours, unless otherwise stated. Images were then analyzed in the open-source software Image J to determine the percentage wound healing at each time point.

### Statistical Analysis

For normally distributed data, a Student’s *t* test was performed, or for grouped data an analysis of variance (ANOVA) was performed. Tukey’s multiple comparisons test was performed post hoc if the ANOVA was deemed significant. For nonparametric data the Mann-Whitney U test was performed, or when there was grouped analysis the Kruskal-Wallis one-way analysis of variance.

## Results

### Identification of Differentially Expressed Genes Associated With Recurrence in PTC

**Figure 1.**
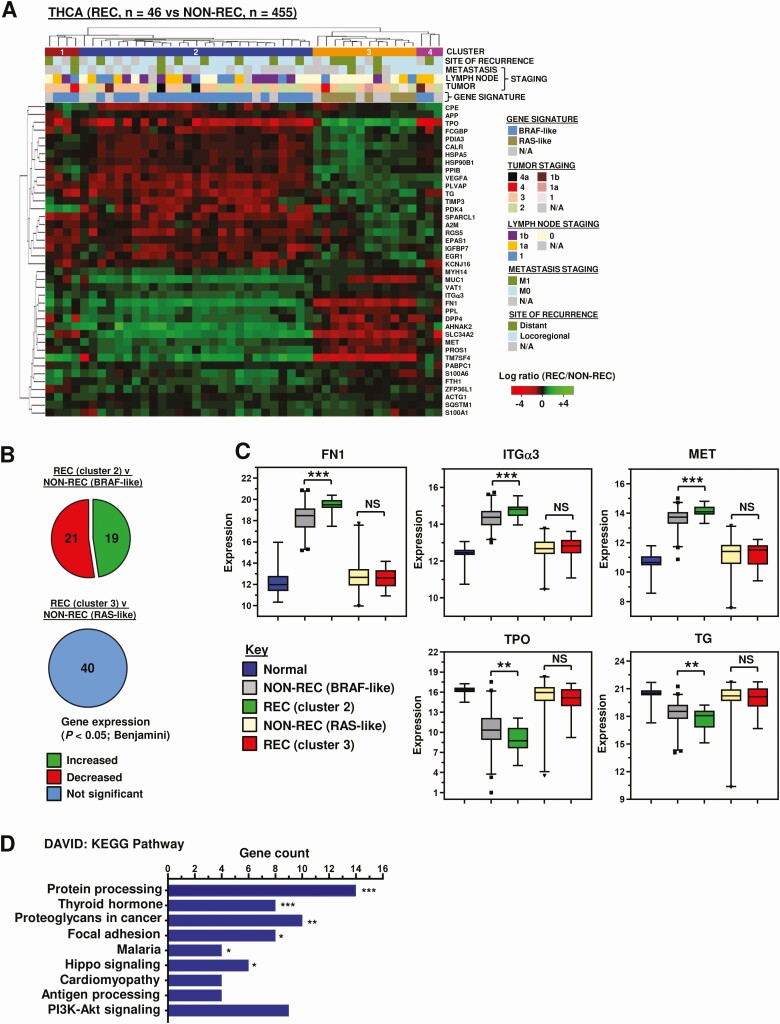
Identification of differentially expressed genes in recurrent PTC with a BRAF-like signature. (A) Hierarchical cluster analysis of the thyroid cancer cohort (THCA, n = 501) based on log_2_ fold change (log_2_FC) (recurrent [REC] vs nonrecurrent [NON-REC]) of top 40 differentially expressed genes. (B) Pie chart showing proportion of significant gene changes in (*upper*) REC (cluster 2, n = 27) (*upper*) vs NON-REC (BRAF-like, n = 245) and (*lower*) REC (cluster 3, n = 12) vs NON-REC (RAS-like, n = 111) THCA cohorts. (C) Box and whisker plots showing expression (log_2_) of 5 genes in BRAF- or RAS-like REC THCA cohorts relative to gene signature-matched NON-REC controls or normals (NS, not significant; **P < 0.01; ***P < 0.001; Mann-Whitney test). (D) Database for Annotation, Visualization and Integrated Discovery (DAVID) functional classification of top 200 differentially expressed genes in REC vs NON-REC patients in THCA. KEGG pathway categories of greatest significance (*P* < 0.05 or lower) and number of genes per category are indicated. (E) Summary of study design to identify and assess the functional impact of biomarkers in recurrent papillary thyroid cancer.

We first appraised TCGA RNAseq data to compare recurrent thyroid cancer patient tumors (THCA; n = 46) vs nonrecurrent (n = 455). Forty genes were significantly different in recurrent disease compared with nonrecurrent tumors from the top 100 median differentially expressed genes (Table S3 (18)). Hierarchical cluster analysis identified 2 major clusters associated with either a BRAF-like (cluster 2) or RAS-like (cluster 3) gene signature (Fig. 1A). These genes remained significantly differentially expressed in recurrent vs nonrecurrent patients in a BRAF-like subset, but not a RAS-like subset (Fig. 1B and 1C; Fig. S3A and S3B (18)). RAS-like tumors were more likely to have a similar RNA expression profile to matched normal tissue (Fig. 1C and Fig. S3C (18)). Within the BRAF-like cluster, 38 of the 40 top differentially expressed genes were also significantly different to expression in normal thyroid tissue (Fig. S3C (18)).

**Figure 2.**
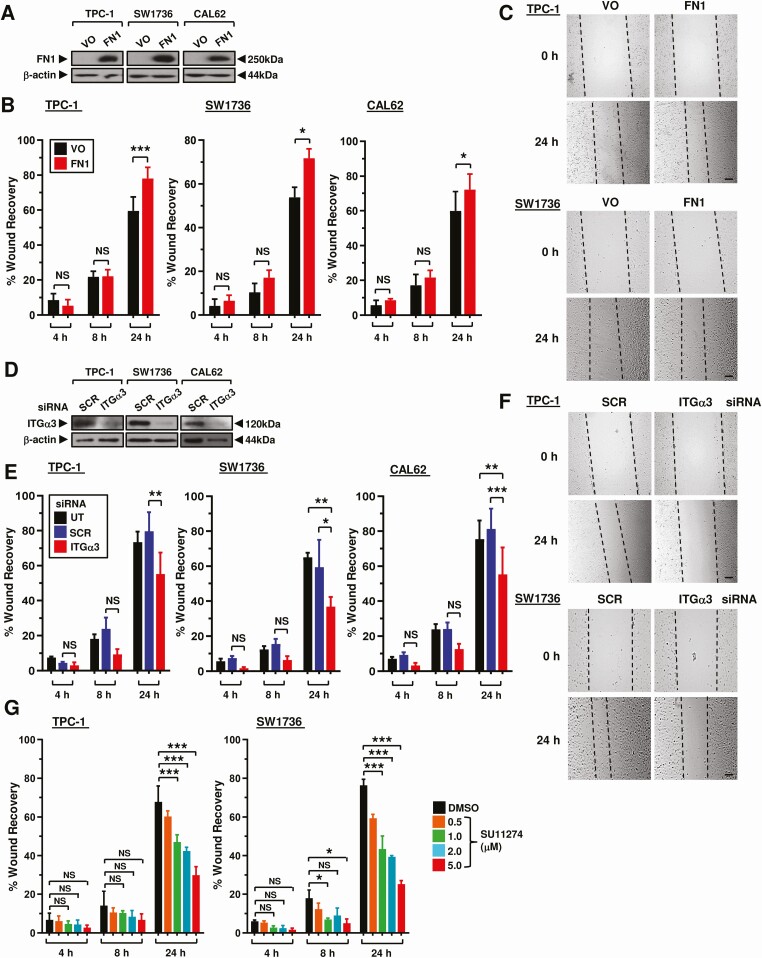
Modulation of genes upregulated in recurrent PTC impact cellular migration. (A) Western blot analysis of FN1 in TPC-1, SW1736, and CAL62 cells transfected with vector-only (VO) or FN1. (B) Quantification of scratch wound assay in TPC-1, SW1736 and CAL62 cells transfected with VO or FN1. %wound recovery determined at indicated time point post-recovery (h). Results expressed as mean ± SEM (NS, not significant; * *P* < 0.05, ***P < 0.001). (C) Representative images of scratch wound assays in TPC-1 (*upper*) and SW1736 (*lower*) cells transfected with VO or FN1 at 24 hours post-recovery. Scale bar, 200 μm. (D) Western blot analysis of ITGα3 in TPC-1, SW1736 and CAL62 cells transfected with ITGα3 or Scr siRNA. (E, F) Same as (B, C) but cells were untransfected (UT) or transfected with ITGα3 or Scr siRNA. Scale bar, 200 μm. (G) Quantification of scratch wound assays in TPC-1 and SW1736 cells treated with SU11274 at the indicated dose (μM). %wound recovery determined at indicated time point post-recovery (h). Data presented as mean ± SEM (NS, not significant; *P < 0.05, ***P < 0.001).

Enrichment analysis highlighted several Kyoto Encyclopedia of Genes and Genomes (KEGG) pathways, including protein processing, thyroid hormone synthesis, focal adhesion, and proteoglycans in cancer (Fig. 1D). FN1, ITGα3, and MET proto-oncogene (MET) were chosen for functional investigation based on their marked expression differences in recurrent vs nonrecurrent BRAF-like PTC (Fig. 1C), and notable prevalence in KEGG pathways (Fig. 1D). Modulation of FN1 and ITGα3 expression levels resulted in significant alteration of cell migration in scratch wound assays. FN1, for instance, induced significant wound healing at 24 hours post-recovery in all 3 thyroid cell lines (Fig. 2A-2C). Conversely, knockdown of ITGα3 was associated with markedly reduced cell migration at 24 hours in all 3 thyroid cell lines (Fig. 2D-2F and Fig. S4A (18)). Targeted inactivation of MET using the well-characterized inhibitor SU11274 gave a significant dose dependent reduction in cell migration in TPC-1 and SW1736 cells, but not in the RAS-mutated CAL62 cells (Fig. 2G; Fig. S4B-4C (18)). Control experiments did not reveal any significant changes in cell proliferation or viability (Fig. S4E and S4F (18)), apart from ITGα3-depletion in CAL62 cells (Fig. S4F (18)).

### Exonic Mutational Analysis Identifies New Gene Variants Associated With Recurrence

Having investigated the impact of 3 genes that showed marked expression differences in recurrent PTC, we next appraised potential mutations that might differentiate recurrent from nonrecurrent tumors. Two somatic exonic mutation pipelines were implemented and called 960 and 2 074 396 variants (TCGA Mutect vcf vs in house TCGA BAM file analysis respectively) which were then filtered and ranked by SIFT and PolyPhen2 scores (Fig. S1B, Tables S4 and S5 (18)). All filtered variants were confirmed as heterozygous mutations found in tumor tissue but not in the matched normal tissue, as visualized in the Integrative Genomics Viewer (35).

**Figure 3.**
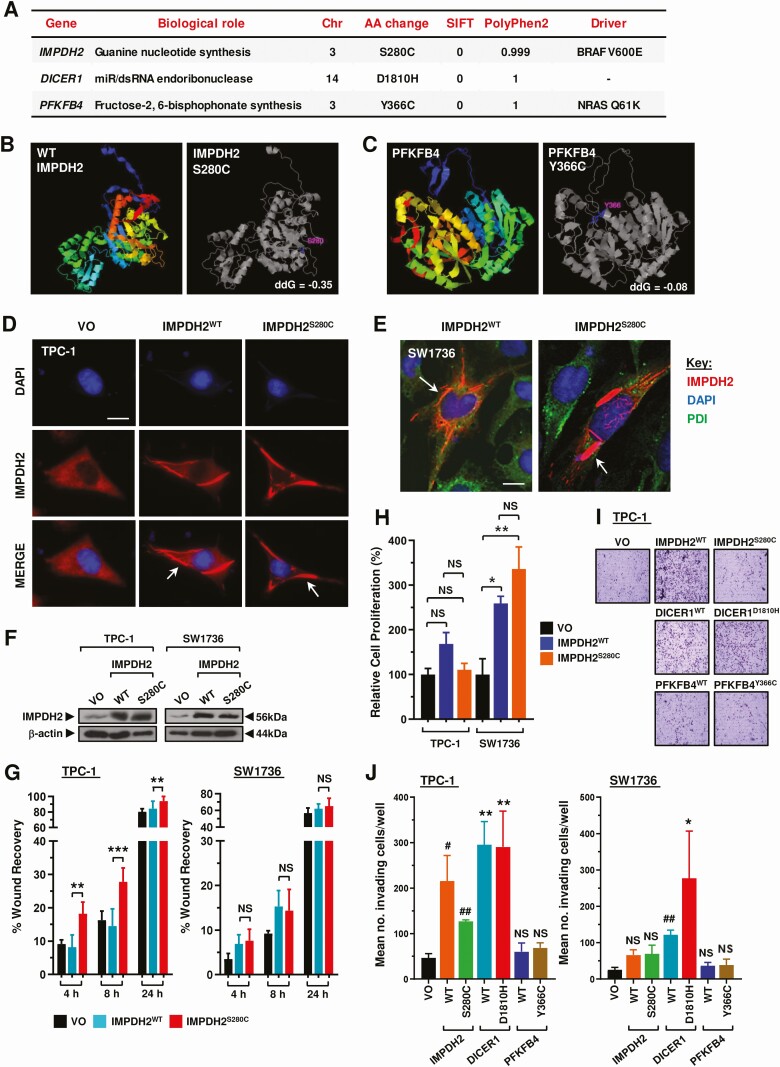
Functional assessment of gene variants identified by exonic mutational pipelines. (A) Three gene variants evaluated in study given their low SIFT and high PolyPhen2 scores. Abbreviations: Chr, chromosome; AA, amino acid; Driver, background driver mutation. (B, C) STRUM structure stability prediction for IMPDH2^S280C^ (B) and PFKFB4^Y366C^ (C). Protein structure predicted by iTASSER with mutation highlighted in gray image (*right*). (D) Representative immunofluorescense images showing characteristic rod and ring (RR) structures (red, white arrow) in TPC-1 cells transfected with IMPDH2^WT^ or IMPDH2^S280C^. Scale bar, 10 μm. (E) Representative confocal images showing RR structures (white arrows) in SW1736 cells transfected with IMPDH2^WT^ or IMPDH2^S280C^. Scale bar, 10 μm. (F) Western blot analysis of IMPDH2 in TPC-1 and SW1736 cells transfected with vector-only (VO), IMPDH2^WT^, or IMPDH2^S280C^. (G) Quantification of scratch wound assay in TPC-1 and SW1736 cells transfected with VO, IMPDH2^WT^, or IMPDH2^S280C^. %wound recovery determined at indicated time point post-recovery (h). Data presented as mean ± SEM (NS, not significant; **P < 0.01, ***P < 0.001). (H) Relative cell proliferation of TPC-1 and SW1736 cells transfected with IMPDH2^WT^ or IMPDH2^S280C^ for 24 hours relative to VO. Results expressed as mean ± SEM (NS, not significant; *P < 0.05, **P < 0.01). (I) Representative images of cell invasion experiments in TPC-1 cells transfected with wild-type (WT) and indicated variants of IMPDH2, DICER1, and PFKFB4 relative to VO. (J) Quantification of invading TPC-1 and SW1736 cells from assays described in (I) relative to VO. Data presented as mean ± SEM, 1-way ANOVA (*P < 0.05; **P < 0.01) or unpaired 2-tailed *t* test (^#^*P* < 0.05; ^##^*P* < 0.01).

Three mutations (IMPDH2^S280C^, PFKFB4^Y366C^, and DICER1^D1810H^) were taken forward for functional investigation given their low SIFT and high PolyPhen2 scores, signifying high likelihood of deleterious effect (Fig. 3A). Well-characterized and expected variants including BRAF^V600E^ ranked highly in our analysis of recurrent thyroid cancers (Table S5 (18)). STRUM structure stability prediction for IMPDH2^S280C^ and PFKFB4^Y366C^ indicated both mutations to be destabilizing and to cause structural change to the proteins (Fig. 3B and 3C).

Transfection of wild-type (WT) and mutant IMPDH2 into TPC-1 and SW1736 cells produced characteristic rod and ring (RR) structures (cytoophidia), which have not been reported previously in thyroid cells (Fig. 3D and 3E). There was no difference in the number or size of cytoophidia between IMPDH2^WT^ and IMPDH2^S280C^ transfected cells (data not shown). IMPDH2^S280C^ significantly increased cellular migration of TPC-1 cells at 4, 8, and 24 hours in scratch wound assays compared with IMPDH2^WT^ (Fig. 3F and 3G; Fig. S5A (18)) but taken as a whole showed no consistent differences from IMPDH2^WT^ in modulating cell proliferation or invasion (Fig. 3H-3J and Fig. S5B). Subsequent screening of DICER1 and PFKFB4 in multiple cell lines did not reveal any functional differences between WT or mutant isoforms in any cellular assay (Fig. 3I and 3J and Fig. S5C-5G (18)).

### miRs-486 and -1179 Are Associated With Recurrence and Influence Cancer Cell Migration

**Figure 4.**
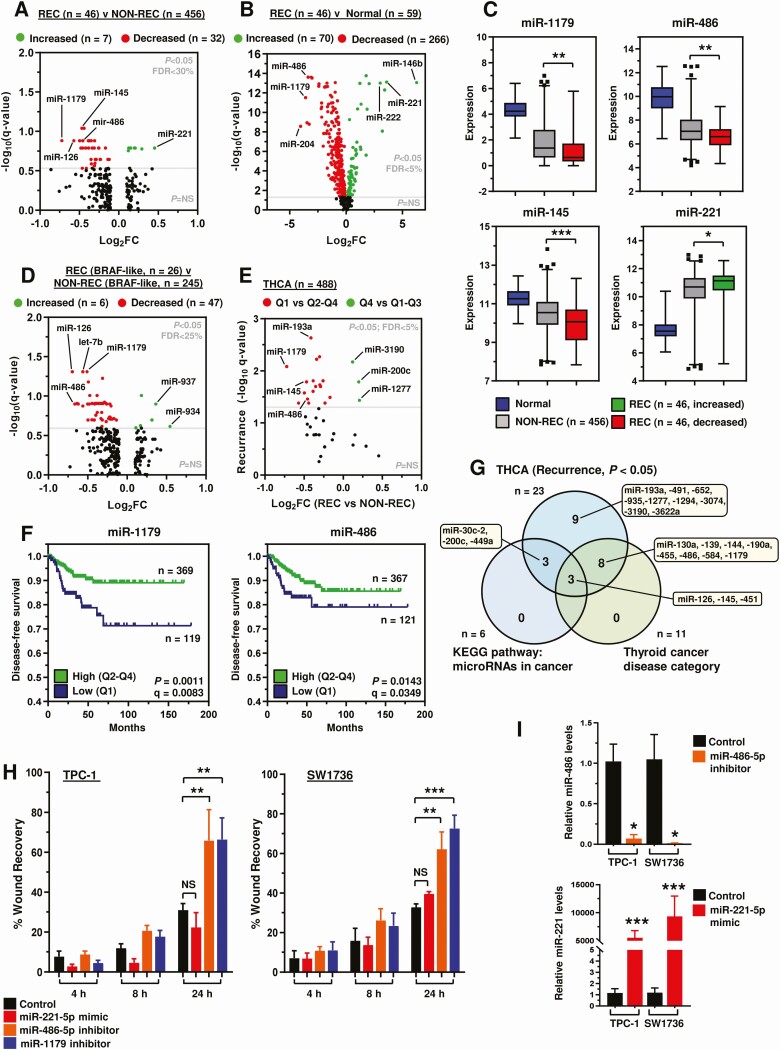
Ablation of miR-486 and miR-1179 increases cellular migration of thyroid cancer cells. (A, B) Volcano plots comparing log_2_ fold change (Log_2_FC) with q-value (-log base 10) for miRNAs in the recurrent (REC) THCA cohort vs the nonrecurrent (NON-REC) THCA cohort (A) or normal (B). miRNAs with log_2_FC ≥ 0.1 or ≤ −0.1 are shown (A, n = 235; B, n = 432). (C) Box and whisker plots showing expression (log_2_) of 4 miRNAs in THCA (REC vs NON-REC) relative to normal. (D) Same as (A) but in the BRAF-like THCA cohort. (E) Volcano plot illustrating log_2_FC in THCA (REC vs NON-REC) compared with q-value (-log base 10) for recurrence (high vs low tumoral expression) for 39 differentially expressed miRNAs [(A), *P* < 0.05]. (F) Representative Kaplan-Meier analysis of disease-free survival for the THCA cohort stratified on high vs low tumoral expression of indicated miRNAs; log-rank test. Number (n) of patients per expression subgroup (high/low), *P* values, and q-values are shown. (G) Venn diagram showing overlap in the categorization of 23 miRNAs associated with recurrence (E, *P* < 0.05) and enriched via gene list analysis (ToppGene: microRNAs in cancer and thyroid cancer disease categories). (H) Quantification of scratch wound assays in TPC-1 and SW1736 cells transfected with miRNA mimic (miR-221-5p) and inhibitors (miR-486-5p, miR-1179). %wound recovery determined at indicated time point post-recovery (h). Data presented as mean ± SEM (NS, not significant; **P < 0.01, ***P < 0.001). (I) Relative miR-486 (*upper*) and miR-221 (*lower*) expression in TPC-1 and SW1736 cells transfected with the indicated miRNA inhibitor or mimic relative to control.

We next investigated whether the expression of miRs might differ between recurrent and nonrecurrent tumors. Our TCGA analysis identified that expression of 39 miRs were significantly different in recurrent tumors (Fig. 4A). By comparison, 336 miRs were dysregulated in recurrent tumor compared with normal thyroid tissue, with 70 overexpressed and 266 underexpressed (Fig. 4B and 4C). Similarly, expression of 53 miRs were dysregulated in a BRAF-like only subset analysis (Fig. 4D and Fig. S6 (18)).

Stratification using quartile expression cutoff values validated the association of dysregulated miRs with recurrence in THCA (Fig. 4E and 4F; 23/39 genes; *P* < 0.05), with similar but distinctive patterns of miRs apparent in BRAF- and RAS-like PTC (Fig. S7 (18)). Pathway analyses identified dysregulated miRs enriched in relevant categories such as miRNAs in cancer and thyroid cancer disease (Fig. 4G and Fig. S8 (18)). Of these, miR-221 has been implicated in thyroid cancer recurrence previously (36), whereas the functional role(s) of other miRs such as miR-486 and -1179 in PTC are less well characterized despite a clear association with recurrence in our study. We selected these 3 miRs (ie, miR-486, miR-1179, and miR-221) to investigate in vitro, based on relative fold-changes in recurrent disease (Fig. 4A-4D), and association with reduced disease-free survival (Fig. 4E and 4F).

Functional analysis showed that ablation of miR-486 and miR-1179 significantly increased the cellular migration of TPC-1 (*P* < 0.01) and SW1736 cells (*P* < 0.001) at 24 hours in scratch wound assays (Fig. 4H and S9A (18)). In contrast, overexpression of miR-221, which is upregulated in recurrent PTC, did not affect cellular migration in either thyroid cell line (Fig. 4H). Control experiments confirmed successful modulation of miR-486, miR-221, and miR-1179 (Fig. 4I and S9B (18)), as well as no significant impact on cellular proliferation (Fig. S9C (18)).

### Clinical Relevance of Validated Biomarkers as a Prognostic Risk Score in Recurrent PTC

**Figure 5.**
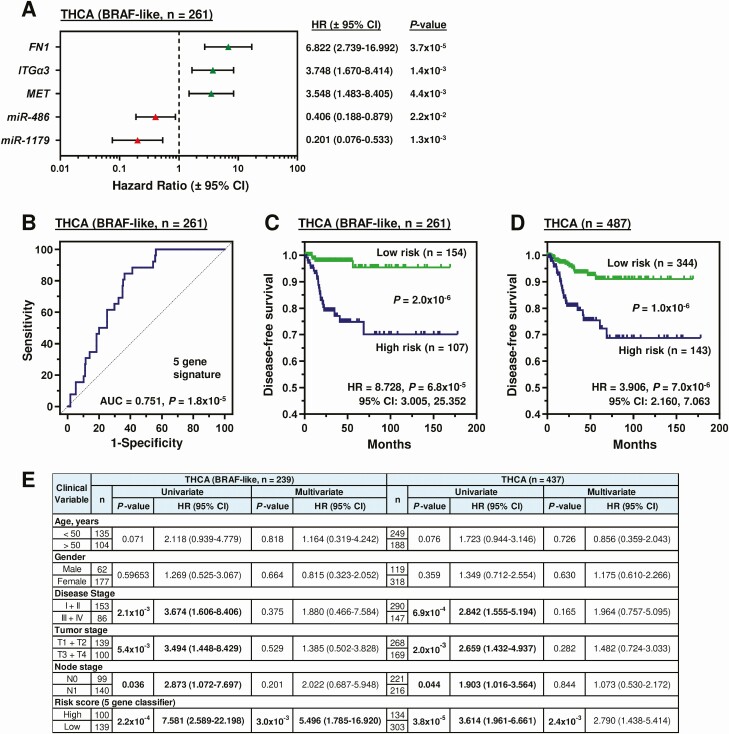
A risk score classifier based on functionally validated miR- and protein-encoding genes is predictive of PTC recurrence. (A) Hazard ratios (HR) ± 95% CI for the BRAF-like THCA cohort stratified using optimal expression cutoff values for indicated genes (univariate Cox regression analysis). (B-D) Receiver operating characteristic (ROC) analysis (B) and Kaplan-Meier curves of the 5-gene risk score signature in the BRAF-like (C) or entire (D) THCA cohort. (E) Univariate and multivariate analysis of the BRAF-like and entire THCA cohorts. Some patients in the BRAF-like (n = 22) and entire THCA (n = 50) cohorts were not included due to missing clinical variables. Abbreviations: n, number; HR, hazard ratio.

Taken as a whole, the genes FN1, ITGα3 and MET, and the miRs miR-486 and -1179 all had significant functional effects in vitro. A ROC curve was therefore generated for each gene/miR to determine the optimized cutoff value for risk stratification (Fig. S10A and S10B (18)). The disease-free survival change was significant for each biomarker using the ROC generated cutoff value to categorize patients into high or low tumoral expression (Fig. S10C (18)). Univariate Cox regression analysis suggested that each biomarker significantly correlated with an increased risk of recurrence (*P* < 0.05; Fig. 5A) in BRAF-like tumors. These genes were therefore combined to construct a prognosis risk model. Somatic mutations (eg, IMPDH2^S280C^) were not assessed for their prognostic utility due to their relative low frequency in recurrent cases.

Risk scores were calculated using multivariate Cox regression coefficient and FPKM expression values (Fig. S10D (18)). Of significance, a higher AUC of 0.751 (Fig. 5B) indicated a greater prediction effect for the 5-gene-based risk score compared with using individual genes (Fig. S10A (18)). Patients indicated as high risk by the combined risk score had a significantly worse recurrence prognosis in the BRAF-like (*P* = 6.8 × 10^−5^; Fig. 5C and Fig. S10E (18)) and entire THCA cohort (*P* = 7.0 × 10^−6^; Fig. 5D).

Importantly, after controlling for age, gender, disease stage, tumor stage, and node status, multivariate analysis showed that the 5-gene risk score classifier was the sole independent prognostic factor for the entire group of THCA patients, as well as for the BRAF-like cohort (Fig. 5E).

## Discussion

Multiple mechanisms are likely to contribute to thyroid cancer recurrence. Increased angiogenesis, escape from the immune system, the cessation of tumor cell dormancy and changes in the extracellular matrix may all be implicated (8, 12, 14, 15, 17, 37, 38). Bioinformatic interrogation of available datasets revealed that at the time of investigation 46 of 501 reported papillary thyroid cancers (PTC) had recurred; our appraisal of gene mutation, gene expression and miR expression revealed that tumor recurrence was not likely to be explained by single somatic mutational events as much as by wider changes to mRNA and miR expression.

Although experimentally we could not consider all the genes which showed significantly altered expression in recurrent vs nonrecurrent tumors, FN1 and ITGα3 both clearly impacted cell migration, hinting at roles which may be related to cellular mechanisms of tumor recurrence. The explanation underlying FN1 upregulation and ITGα3 downregulation in those tumors which subsequently recurred is not clear, but a previous study aimed at understanding the aggressive nature of BRAF-driven tumors identified—among others—ITGα3 and FN1 as showing marked dysregulation (39). FN1 has a role in cell adhesion and cell motility, upregulation of which would potentially generate the ability for tumors to both metastasize and recur. FN1 has also been linked with cancer aggressiveness previously, both generally and in the context of thyroid cancer (40-43). Fibronectin is also a marker of mesenchymal status in the epithelial mesenchymal transition, part of the accepted development of cells from primary tumor to invasion, metastasis, and recurrence (44, 45). Previous studies have demonstrated an association between FN1 expression and the invasive, migratory, and proliferative nature of thyroid cancer cell lines (40, 46). What is especially important here from the TCGA data is that the increased FN1 level was apparent at the patients’ initial histology, highlighting this as a potential prognostic biomarker.

Interestingly, FN1 and ITGα3 are able to influence each other’s expression (47), interacting directly under certain conditions, with fibronectin binding to ITGα3/β1 (48, 49) and ITGα3/β1 increasing the deposition of fibronectin into the pericellular matrix (50). Integrin α3 is present in invadopodia in breast cancer cells (51) and recruits extracellular matrix (ECM) degrading proteases (52, 53). This breakdown of the ECM helps tumor cells invade and even metastasize. Integrins are not only involved in tumor invasiveness, but also are implicated in cancer cell dormancy after treatment and consequent recurrence (54). The activation of ITGα3/β1 causes phosphorylation of FAK, leading to increased kinase activity, including interaction with Src and cortactin (54).

In addition to FN1 and ITGα3, KEGG pathway analysis highlighted cell adhesion and ECM organization, as well as endoplasmic reticulum protein processing, as key pathways dysregulated in recurrent vs nonrecurrent PTC. Cell adhesion, focal adhesion, and ECM interactions have all previously been implicated in recurrent disease. The association with recurrence originates from implicating integrins in the switch to cancer cell dormancy, treatment evasion and cooperative signaling with receptor tyrosine kinases for cell survival (54). Proteoglycans in cancer came up in both arms of the analysis. This encompasses both MET and the MAPK and PI3K-Akt pathways, all frequently implicated in thyroid cancer progression and aggressiveness.

One surprise of the current study was that specific mutations in genes we identified (IMPDH2^S280C^, PFKFB4^Y366C^, and DICER1^D1810H^) which had predicted likelihoods of highly deleterious effects did not markedly alter the cell behaviors we assessed. Functionally, IMPDH2 is the rate-limiting enzyme in guanine nucleotide synthesis and has been associated with tumor progression (55), as well as cellular proliferation and tumorigenesis (56). PFKFB4 regulates glycolysis by synthesis of fructose-2,6-bisphosphate (57), aids malignant cells evade apoptosis, and high expression is associated with multiple different malignancies (58). DICER1 encodes the RNAse III enzyme integral to small interfering RNA (siRNA) gene regulation, being a critical component of the RNA-induced silencing complex (RISC), which loads siRNA and miRNA onto mRNA, facilitating their posttranscriptional gene regulation (59). The IMPDH2^S280C^ mutation appeared in a BRAF^V600E^-mutated patient with a subsequently recurrent thyroid cancer, and the PFKFB4^Y366C^ mutation occurred on a background NRAS^Q61K^ mutation, whereas the DICER1^D1810H^ mutation occurred in a patient with no BRAF/RAS mutation. Additional mutations, particularly in the presence of BRAF^V600E^, are important and can dictate prognosis (60). Although our cellular studies attempted to broadly mimic these background mutations, it is possible that other genetic hallmarks of the dedifferentiated cell lines used may have masked any impact of cell invasion, migration, and proliferation. Alternatively, activation of the canonical pathways of thyroid cell dedifferentiation rendered these mutations—despite having high probabilities of being deleterious—not functionally dominant in the assays used to appraise them, especially in CAL62 and SW1736 cell lines which are particularly dedifferentiated.

The expression of miRs is known to be corrupted in PTC compared to normal thyroid tissue (19). Our unique angle was to consider this through the lens of early tumor recurrence. Multiple miRs showed significantly altered expression in tumors which subsequently recurred compared to those which did not. We investigated 3 of these functionally (miR-221, miR-486, and miR-1179), mimicking the direction of their expression changes in recurrent tumors. This revealed that miR-486 and miR-1179 knockdown was associated with significantly enhanced cell migration. miR-486 was of interest as it has been demonstrated to be underexpressed in differentiated thyroid cancer (61) and associated with cancer stage, survival, and recurrence (62). Similarly, miR-1179 is underexpressed in thyroid cancer (61, 63), and in the current study was the most differentially expressed miR in terms of median differential expression. One study identified both miR-476 and miR-1179 as potential biomarkers for thyroid cancer but did not associate them with recurrence (64). A key difference was that the study by Rosignolo et al used clinical factors to predict risk of recurrence, whereas here the available TCGA data indicated patients with confirmed instances of thyroid cancer recurrence.

An important finding was that miR-486 and miR-1179 demonstrated significantly increased wound healing in 2 thyroidal cell lines, indicating that they may not just be “passengers” with reduced expression in thyroid cancer recurrence, but have an active role in oncogenesis. miR-486 has multiple gene regulatory targets (65), including the extracellular matrix protein fibrillin-1 (66). In functional investigations of miR-1179, one study found that the circular RNA hsa_circ_0039411 affected cell growth, migration, and invasion by acting as a sponge for miR-1179 in PTC (67). It is thus likely that the role of miR-1179 in PTC is complex and may influence multiple mechanisms which impact on tumor recurrence.

In the current study, then, we assessed 3 levels of genetic change that might predispose some thyroid cancers to early recurrence. Collectively, we have performed a full appraisal of the next-generation sequencing and TCGA datasets for recurrent PTC and identified new biomarkers for tumor recurrence. We show that tumor recurrence is less likely to be explained by single mutational events than by wider changes to mRNA and miR expression profiles in recurrent vs nonrecurrent PTC, and how these changes may be critical to tumor recurrence prediction. Importantly, we validated functional roles for 5 biomarkers in modulating features associated with tumor aggressiveness. We propose that this risk score is therefore an actionable prognostic indicator of PTC recurrence, based on expression values observed in patients’ initial tumor samples. One drawback of the risk score would be the additional time and potential cost this might add to the patient journey, with a clinical evaluation of the cost-benefit impact required before this could be truly considered for implementation.

Our risk score classifier clearly showed that a combination of miRNA and protein-encoding genes has potential adaptation for clinical use in prediction of recurrence rates. We envisage that patients could be categorized further in terms of risk of recurrence by using these gene expression values from the initial tumor, after their original diagnostic or therapeutic surgery. With the limited treatment options available (5, 6), early prediction of recurrent disease should impact favorably on patient outcomes by tailoring treatment to disease risk and increasing posttreatment recurrence surveillance. Our data suggest that the expression of genes FN1, ITGα3, MET, miR-486, and miR-1179, can be useful future prognostic tools in indicating the likelihood of individual PTC recurrence.

## Data Availability

Some of the data generated or analyzed during this study are included in this published article or in the data repositories listed in References (Supplementary figures). The rest of the datasets generated and/or analyzed during the current study are not publicly available but are available from the corresponding author on reasonable request.

## Abbreviations

ANOVA: analysis of variance
ECM: extracellular matrix
FBS: fetal bovine serum
FN1: fibronectin 1
IMPDH2: inosine monophosphate dehydrogenase 2
ITGα3: integrin α3
KEGG: Kyoto Encyclopedia of Genes and Genomes
Log_2_FC: Log_2_ fold change
MET: MET proto-oncogene, receptor tyrosine kinase
miR: microRNA
PFKFB4: 6-phosphofructo-2-kinase/fructose-2,6-bisphosphatase 4
PolyPhen2: polymorphism phenotyping version 2
PTC: papillary thyroid cancer
REF: reference base
ROC: receiver operating characteristic
RR: rod and ring
SIFT: sorting intolerance from tolerance
siRNA: small interfering RNA
SS: somatic score
TCGA: The Cancer Genome Atlas
THCA: thyroid cancer
vcf: variant call format
WT: wild-type

## Additional Information

***Disclosures:*** The authors have nothing to disclose.
